# Prediction of distant organ metastasis and overall survival of lung cancer patients: a SEER population−based cohort study

**DOI:** 10.3389/fonc.2023.1075385

**Published:** 2023-06-12

**Authors:** Yongping Hao, Guang Li

**Affiliations:** Department of Radiation Oncology, The First Affiliated Hospital of China Medical University, Shenyang, Liaoning, China

**Keywords:** lung cancer, distant metastasis, risk factor, prognosis, SEER

## Abstract

**Background:**

Distant organ metastasis is a common event in lung cancer (LC). However, the preferential metastatic pattern of different pathological types of LC and its effect on prognosis have not been comprehensively elucidated. This study aimed to explore the distant metastasis pattern and construct nomograms predicting the metastasis and survival of LC patients using the Surveillance, Epidemiology, and End Results (SEER) database.

**Methods:**

LC data were downloaded from the SEER database to conduct logistic regression and investigate risk factors for developing organ metastasis. A Cox regression analysis was conducted to investigate prognostic factors of LC. A Kaplan–Meier analysis was used to estimate overall survival outcomes. Nomograms were constructed to predict the probability of organ metastasis and the 1-, 3- and 5-year survival probability of LC patients. Receiver operating characteristic curves were used to evaluate the diagnostic accuracy of the nomograms. All statistical analyses were conducted within R software.

**Results:**

The liver is the most common metastatic organ of small cell carcinoma. The brain is the most likely metastasis site of large cell carcinoma, and bone is the most likely metastasis site for squamous cell carcinoma and adenocarcinoma. Patients with triple metastases (brain-bone-liver) have the worst prognosis, and for nonsquamous carcinoma with single organ metastasis, liver metastases conferred the worst prognosis. Our nomograms based on clinical factors could predict the metastasis and prognosis of LC patients.

**Conclusion:**

Different pathological types of LC have different preferential metastatic sites. Our nomograms showed good performance in predicting distant metastasis and overall survival. These results will provide a reference for clinicians and contribute to clinical evaluations and individualized therapeutic strategies.

## 1 Introduction

According to the estimation, there will be 236740 new cases of lung cancer in the USA in 2022, with 130180 deaths. LC is the second most common cancer in both men and women, less than prostate cancer (in males) and breast cancer (in females), and LC is the leading cause of death among cancer patients, with a low 5-year survival rate (1). Metastasis is a characteristic of cancer and is responsible for the greatest number of cancer-related deaths (2). Approximately 20% of cancer patients will develop brain metastases(BMs) (3), and brain metastases from LC account for approximately 45% of the total cases of BMs (4, 5). Approximately 10% of SCLC patients have brain metastases at the time of the initial diagnosis (6). In addition to the brain, the liver and bone are also common metastasis sites of lung cancer (7, 8). Despite the rapid development of multiple therapies, such as targeted therapies and immunotherapy (9), the prognosis of patients with advanced lung cancer remains poor (3). The median survival time of BM patients is approximately 10.6 months (9). It is very important to identify risk and prognostic factors, evaluate individual metastatic risk and make accurate diagnoses to improve the survival outcome. Providing individualized treatment for different patients to maximize personal survival benefits is a research direction (10).

The survival rates for LC patients with different distant organ metastases are not the same. Understanding the epidemiology of the most common distant organ metastasis patterns in different pathological types of LC, as well as their overall survival, will help the process for clinical decisions. A previous study suggested that for SCLC patients with brain metastasis, male sex, older age, liver metastasis, and insurance status were associated with increased death risk (11). For NSCLC patients, age, race, sex, pathology, T stage and N stage were associated with the occurrence of brain metastasis and overall survival (12–14). However, few studies have compared the survival risk among LC patients with different distant metastases and focused on the prediction of distant metastases. The purpose of our study was to describe a detailed landscape of distant organ metastasis status and explore the effects of distant organ metastasis status on overall survival in different pathological types of lung cancer. We also analyzed the risk factors for organ metastasis and prognostic factors in LC patients based on data from the Surveillance, Epidemiology, and End Results (SEER) database (15). Moreover, we tried to construct nomograms predicting the organ metastasis and overall survival of LC patients.

## 2 Methods

### 2.1 Population

**Figure 1:**
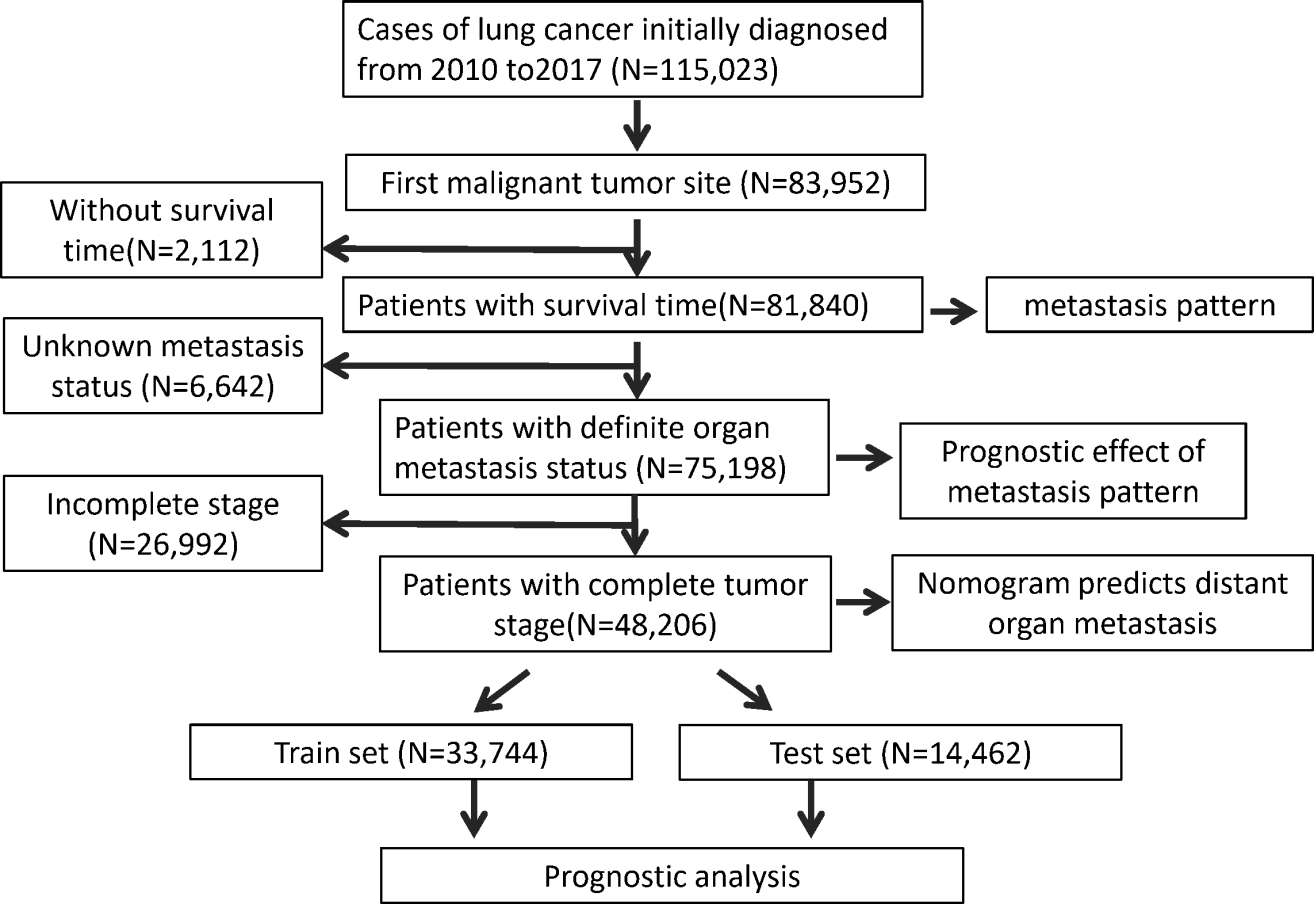
Flowchart of the process of data selection.

In this population-based study the LC patient data were downloaded from the SEER*Stat Database: Incidence - SEER Research Plus Data. SEER*Stat version 8.4.0 (https://seer.cancer.gov/seerstat/) was used to obtain the patient information (15). The extraction condition was “the site of the tumor: lung”. The following variables were extracted: Age recode; Race recode;patient ID, Sex, Year of diagnosis, Primary Site, ICD-O-3 Hist/behav, Laterality, Separate Tumor Nodules Ipsilateral Lung Recode; SEER Combined Mets at DX-bone; SEER Combined Mets at DX-brain; SEER Combined Mets at DX-liver; Mets at DX-Distant LN; Survival months; Vital status recode; 8th edition AJCC classification; Sequence number. LC patients who were diagnosed between 2010 and 2017 were included in this study. Patient information was excluded when the lung was not the first primary site. Patient information with survival time was used to explore the metastasis pattern of the LC patients. The data with a definite metastasis status were used to evaluate the prognostic effect of metastasis pattern. After excluding the data without a tumor stage, we investigated the risk factors for developing organ metastasis and the prognostic factors for LC patients and thus constructed the prediction nomogram. The inclusion and exclusion process is shown in **Figure 1**.

### 2.2 Statistical analysis

This study included the following variables: age (<50, 50–59, 60–69, 70–79,>=80); sex (male and female); race (white, black, other (American Indian/AK Native, Asian/Pacific Islander), unknown); pathology (adenocarcinoma, non-small cell lung carcinoma, small cell carcinoma, large cell carcinoma, squamous cell carcinoma, other); site of primary tumor (left main bronchus, left upper lobe, left lower lobe; center main bronchus; center upper lobe; center middle lobe; center lower lobe; other); separate tumor nodule (Yes, No, unknown);T stage (T0, T1, T2, T3, T4, unknown); N stage (N0, N1, N2,N3, unknown); liver metastasis (Yes, No, unknown); bone metastasis (Yes, No, unknown); brain metastasis (Yes, No, unknown). The site of the primary tumor was determined according to “laterality” and “primary site”. Pathology with ICD-O-3 including 8070/8071/8072/8073/8074/8075/8084 was classified as SCC, ICD-O-3 8012/8013/8014 was classified as LCC, ICD-O-3 8040 was classified as AD, ICD-O-3 8046 was classified as NSCLC-NOS, and ICD-O-3 8002/8041/8042/8043/8044/8045 was classified as SCLC. Other variables are directly obtained from the SEER database.

All statistical analyses were conducted within R software (version 4.1.0). Univariate and multivariate logistic regression were used to identify the risk factors for distant organ metastasis. The factors that significantly associated with organ metastasis in univariate logistic regression were included in the following multivariate logistic regression. The Kaplan–Meier (KM) method was used to investigate overall survival outcomes using the log-rank test. Univariate and multivariate Cox regression analyses were used to identify potential prognostic factors for LC patients. The “rms” package was used to construct nomograms that predict the probability of organ metastasis and the 1-, 3- and 5-year survival probability of LC patients. The concordance index (c-index) was calculated to assess the prognostic performance of the nomogram based on the “survival” package. Receiver operating characteristic (ROC) curves were used to evaluate the diagnostic accuracy of the nomograms, which was achieved by the “pROC” package (16). The area under the curve (AUC) was related to the accuracy of the nomogram. P<0.05 was considered to be statistically significant.

## 3 Results

### 3.1 Effects of distant metastasis on patient survival

The brain, bone, and liver are common organs involved in distant metastasis of lung cancer. Therefore, we conducted a subgroup analysis based on pathological types to explore the effect of different distant metastasis modes on LC patient survival. There were a total of 81,840 patients with survival data, consisting of 15,489 SCC patients, 31,100 AD, 10,037 SCLC patients, 5,170 NSCLC-NOS patients, 1,041 LCC patients and 19,003 patients with other types of LC. A total of 6642 patients had unknown organ metastases. The results showed that the probability of distant metastasis was highest in SCLC and lowest in SCC. The liver (14.4%) is the most common single metastatic organ of SCLC, and approximately 10.04% of SCLC patients develop both liver and bone metastases. On the other hand, the brain is the most likely metastasis site for LCC. Moreover, bone is the most common metastatic organ for SCC and AD. The number of patients included in each subgroup is shown in **Table 1**.

**Table 1:** Number of patients included in subgroup analysis.

Pathology	Number of patients									
	total	No (%)	brain (%)	bone (%)	liver (%)	brain+bone (%)	brain+liver (%)	bone+liver (%)	brain+bone+liver (%)	excluded (%)
Squamous cell caicinoma	15489	12094 78. 08%	532 3.43%	1096 7.08%	400 2.58%	167 1.08%	71 0.46%	359 2.32%	97 0.63%	673 4.35%
Adenocarcinoma	31100	18218 58. 58%	2827 9.09%	3995 12.85%	778 2.50%	1486 4.78%	282 0.91%	1133 3.64%	677 2.18%	1704 5.48%
Non-small cell carcinoma-NOS	5170	2828 54.70%	539 10.43%	665 12.86%	210 4.06%	189 3.66%	67 1.30%	227 4.39%	88 1.70%	357 6.91%
Small cell carcinoma	10037	4397 43.81%	888 8.85%	821 8.18%	1449 14.44%	221 2.20%	245 2.44%	1008 10.04%	282 2.81%	726 7.23%
Large cell carcinoma	1041	603 57.93%	106 10.18%	81 7.78%	65 6.24%	31 2.98%	19 1.83%	58 5.57%	21 2.02%	57 5.48%
Other	19003	11915 62.70%	923 4.86%	1179 6.20%	790 4.16%	266 1.40%	138 0.73%	506 2.66%	161 0.85%	3125 16.44%

Then, we performed a Cox analysis to explore the impact of different metastasis statuses on the prognosis of patients. The subgroup analysis suggested that for all pathologic types, the patients without distant metastasis had a better prognosis than the patients with brain metastasis alone, and the patients with brain plus liver metastasis had a worse prognosis than the patients with brain metastasis alone. For SCC, the prognosis of the patients with a single brain metastasis was similar to that of the patients with a single liver metastasis, while the prognosis of the patients with a single bone metastasis was worse. For AD, the survival time of the patients with single bone or single liver metastasis was shorter than that of the patients with single brain metastasis. Among the other pathologic types of lung cancer, single liver metastases were associated with the worst prognosis compared with single brain or single bone metastases, and there was no significant difference in survival between single bone and single brain metastases. Among lung SCC, the patients with triple metastases (brain-bone-liver) had the worst prognosis. For the patients with more than one organ metastasis, bone-liver metastasis in AD and LCC and brain-liver metastasis in NSCLC-NOS and SCLC are associated with the shortest survival (**Table 2**).

**Table 2:** Cox analysis revealed the prognosis of patients with different organ metastases.

Pathology	Distant metastases sites								
Squamous cell carcinoma	metastases HR (95%CI) P value	Brain 1 (reference) 1 (reference)	No 0.336(0.3073-0.3675) <0.001	bone 1.113(1.0023-1.237) 0.045238	liver 0.9116-1.1866) 0.55909	brain+bone 1.406(1. 1804-1.675) <0.001	brain+liver 1.439(1.1225-1.8437) 0.004074	bone+liver 1.35(1.1789-1.5461) <0.001	brain+bone+liver 1.785(1.4351-2.2195) <0.001
Adenocarcinoma	metastases HR (95%CI) P value	brain 1 (reference) 1 (reference)	No 0.4567(0.4373-0.477) <0.001	bone 1.1839(1.1253-1.246) <0.001	liver 1.5116(1.392-1.641) <0.001	brain+bone 1.2096(1.1327-1.292) <0.001	brain+liver 1.587(1.3988-1.8 801) <0.001	bone+liver 1.7215(1.6034-1.848) <0.001	brain+bone+liver 1.5787(1.4481-1.721) <0.001
Non-small cell carcinoma-NOS	metastases HR (95%CI) P value	brain 1 (reference) 1 (reference)	No 0.5306(0.4819-0.5841) <0.001	bone 1.1016(0.9811-1.237) 0. 10156	liver 1.2563(1.0673-1.4788) 0.00608	brain+bone 1.1698(0.9876-1.3858) 0.06952	brain+liver 1.8444(1.4269-2.384) <0.001	bone+liver 1.3962(1.1922-1.635) <0.001	brain+bone+liver 1.357(1.0774-1.7091) 0.0095
Small cell carcinoma	metastases HR (95%CI) P value	brain 1 (reference) 1 (reference)	No 0.5918(0.549-0.6379) <0.001	bone 1.0371(0.9409-1.1431) 0. 46344	liver 1.5683(1.4402-1.7078) <0.001	brain+bone 1.2634(1.0874-1.468) 0.00226	brain+liver 1.7331(1.5022-1.9994) <0.001	bone+liver 1.6116(1.4699-1.7669) <0.001	brain+bone+liver 1.6131 (1.4088-1.8471) <0.001
Large cell carcinoma	metastases HR (95%CI) P value	brain 1 (reference) 1 (reference)	No 0.374 (0.2999-0.4664) <0.001	bone 1.188 (0.8862-1.5923) 0. 249288	liver 1.559(1.1404-2.1319) 0.005383	brain+bone 1.145(0.7575-1.73) 0.520945	brain+liver 1.694(1.0368-2. 7671) 0.035355	bone+liver 1.861(1.344-2.5773) <0.001	brain+bone+liver 1.442(0.9006-2.3086) 0.127512
Other	metastases HR (95%CI) P value	brain 1 (reference) 1 (reference)	No 0.2809(0.2614-0. 3019) <0.001	bone 1.0948(1.0017-1.1965) 0.0459	liver 1.5279(1.3861-1.6843) <0.001	brain+bone 1.0516(0.9143-1.2096) 0.481	brain+liver 1.4595(1.2165-1.751) <0.001	bone+liver 46(1.3067-1.6312) <0.001	brain+bone+liver 1.4675(1.2378-1.7398) <0.001

### 3.2 Predictive factors and nomogram for distant organ metastases in patients with LC

Since distant organ metastasis of LC patients is closely related to prognosis, we tried to screen the clinical factors that can predict organ metastasis and to establish a prediction model. After excluding patients with incomplete stage information, a total of 48,206 patients were enrolled in the following analysis. Of these patients, 8,391 (17.4%) patients were older than 80 years old. A total of 24,956 (51.8%) patients were male. Over half of the patients were white (79.4%, N= 38264). For the lesion site, the center upper lobe was the most common, at approximately 30.2% (N=14548). A total of 24.5% of the patients were diagnosed with separate tumor nodules. Most patients did not have bone metastases (N=39234, 81.4%), liver metastases (N=42875, 88.9%), or brain metastases (N=41858, 86.8%); 68.5% of the patients had no distant organ metastases; and 21.8% of the patients had one single organ metastasis. The cohort of 48,206 patients was divided into a train set (N=33,744) and a test set (N=14,462), with a ratio of 7:3. More details about the clinical characteristics are shown in **Table 3**.

**Table 3:** Clinical characteristics of lung cancer patients.

N	Clinical characteristics of LC patients			
	Overall	train set	test set	p value
	48206	33744	14462	
Age (%)				0.46
<50	2020 (4. 2)	1395(4.1)	625(4.3)	
50-59	7988 (16. 6)	5587(16.6)	2401(16. 6)	
60-69	15130 (31.4)	10573(31.3)	4557(31.5)	
70-79	14677 (30. 4)	10351 30. 7)	4326(29.9)	
>=80	8391 (17. 4)	5838 (17.3)	2553(17.7)	
Race (%)				0.648
Black	4405 (9. 1)	3092 9. 2)	1313(9.1)	
White	38264 (79. 4)	26817(79.5)	11447 (79. 2)	
Other	5463(11.3)	3784(11.2)	1679(11.6)	
Unknown	74(0. 2)	51(0.2)	23(0.2)	
Sex (%)				0.085
Female	23250 (48. 2)	16362(48. 5)	6888(47.6)	
Male	24956 (51. 8)	17382(51.5)	7574(52.4)	
Pathol ogy (%)				0.534
AD	18800 (39. 0)	13239(39.2 2)	5561(38.5)	
SCC	10044 (20. 8)	6980 (20. 7)	3064 (21. 2)	
LCC	668 1. 4)	476(1.4)	192(1. 3)	
NSCLC-NOS	3351 (7. 0)	2351 (7.0)	1000(6.9)	
SCLC	5772(12. 0)	4012(11.9)	1760(12.2)	
other	9571 (19. 9)	6686(19.8)	2885(19.9)	
Site of the primary tumor	(%)			0.561
LLL	5730(11. 9)	4054(12.0)	1676(11. 6)	
LMB	886(1. 8)	616(1. 8)	270(1. 9)	
LUL	11343(23.5)	7989(23.7)	3354(23. 2)	
RLL	7337 (15.2)	5125(15.2)	2212(15. 3)	
RMB	1196 (2.5)	846(2.5)	350 2. 4)	
RML	2289 (4.7)	1598(4.7)	691 (4. 8)	
RUL	14548 (30. 2)	10152(30.1)	4396(30.4)	
other	4877 (10. 1)	364(10. 0)	1513 (10. 5)	
Separate tumor nodule (%)				0.399
NO	34437 (71. 4)	24164(71.6)	10273(71.0)	
YES	11789 (24. 5)	8194(24.1 3)	3595(24. 9)	
Other	1980(4.1)	1386(4. 1)	594(4.) 1)	
Bone metastasis (%)				1
NO	39234 (81. 4)	27464(81.4)	11770(81.4)	
Yes	8972(18.6 6)	6280(18.6)	2692(18.6)	
Liver metastasis (%)				0.523
NO	42875 (88. 9)	30033 (89. 0)	12842(88.8)	
Yes	5331 (11. 1)	3711 (11. 0)	1620(11. 2)	
Brain metastasis (%)				0.346
NO	41858 (86. 8)	29333 (86. 9)	12525(86.6)	
Yes	6348(13.2)	4411 (13. 1)	1937(13. 4)	
T stage (%)				0.364
TO	309 (0. 6)	207 (0. 6)	102(0. 7)	
T1	11170 (23. 2)	7883(23. 4)	3287(22. 7)	
T2	14753 (30. 6)	10283 (30. 5)	4470(30.9)	
T3	10513 (21. 8)	7380(21.9)	3133(21.7)	
T4	11461 (23. 8)	7991 (23. 7)	3470(24.0)	
N stage (%)				0.159
NO	19456 (40. 4)	13730 (40. 7)	5726(39.6) 6)	
N1	4468 9. 3)	3120(9. 2)	1348(9. 3)	
N2	17062 (35. 4)	11866 (35. 2)	5196(35.1 9)	
N3	7220 (15. 0)	5028 (14. 9)	2192(15.2)	
Number of organ etastases				0.682
0	33021 (68. 5)	23125(5)	9896(68. 4)	
1	10529 (21. 8)	7393(21.9)	3136(21.7)	
2	3846 (8. 0)	2669(7.9)	1177(8.1 1)	
3	810(1. 7)	557(1 7)	253 (1. 7)	

SCC, squamous cell carcinoma; AD, adenocarcinoma; SCLC, small cell lung carcinoma; NSCLC-NOS, non-small cell lung carcinoma-not otherwise specified; LCC, large cell carcinoma; LLL, left lower lobe; LMB, left main bronchus; LUL, left upper lobe; RLL, center lower lobe; RMB, center main bronchus; RMB, center middle lobe; RUL, center upper lobe.

**Figure 2:**
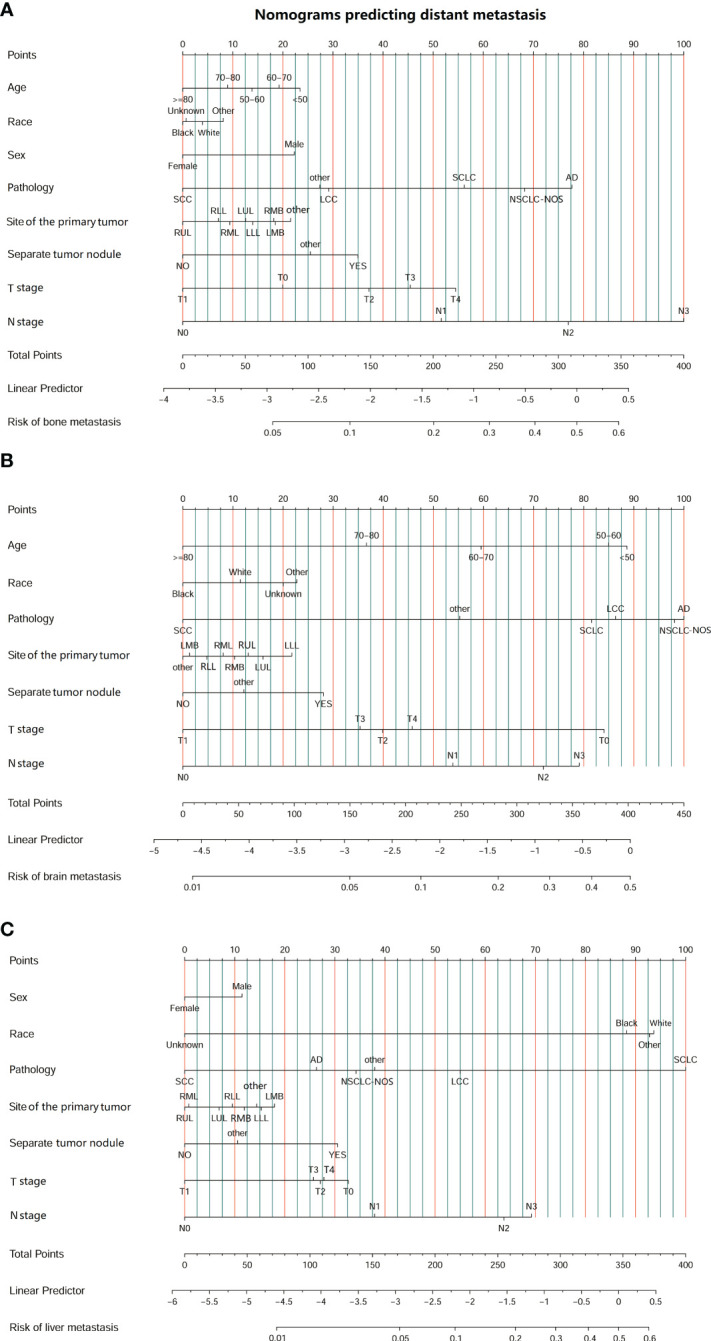
Nomograms predicting the risk of organ metastasis in patients with lung cancer. **(A)** Nomogram predicting the risk of bone metastasis in patients with LC. **(B)** Nomogram predicting the risk of brain metastasis in patients with LC. **(C)** Nomogram predicting the risk of liver metastasis in patients with LC. (SCC, squamous cell carcinoma; AD, adenocarcinoma; SCLC, small cell lung carcinoma; NSCLC-NOS, non-small cell lung carcinoma-not otherwise specified; LCC, large cell carcinoma; LLL, left lower lobe; LMB, left main bronchus; LUL, left upper lobe; RLL, right lower lobe; RMB, right main bronchus; RMB, right middle lobe; RUL, right upper lobe.).

We conducted univariate and multivariate logistic regression analyses to analyze the risk factors for distant organ metastasis in patients with LC. The results showed that age, race, sex, pathology, site, separate tumor nodule, T stage, and N stage were related to bone metastasis (**Supplementary Table 1**). Age, race, pathology, site, separate tumor nodule, T stage, and N stage were related to brain metastasis (**Supplementary Table 2**). Race, sex, pathology, site, separate tumor nodule, T stage, and N stage were related to liver metastasis (**Supplementary Table 3**). Then, these predictive clinical factors were used to construct nomograms to predict distant metastasis of the bone, brain and liver. The predictive model was constructed based on the train set and was verified in the test set. The nomograms are shown in **Figure 2**. The total points, based on the calculation of each variable point, were associated with the probability of organ metastasis.

**Figure 3:**
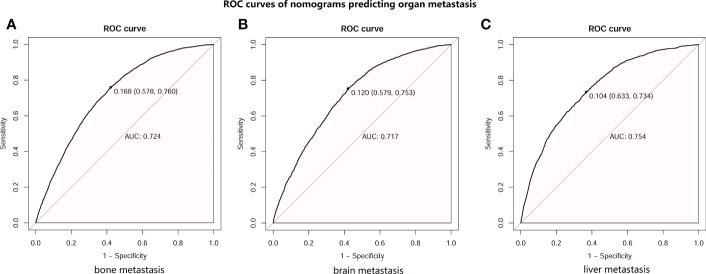
ROC curves of distant metastasis prediction nomograms in patients with lung cancer (LC). **(A)** ROC curve of nomogram predicting bone metastasis in patients with LC. **(B)** ROC curve of nomogram predicting brain metastasis in patients with LC. **(C)** ROC curve of nomogram predicting liver metastasis in patients with LC.

Then, an ROC curve was constructed using the test set to assess the accuracy of the nomogram in predicting the development of distant organ metastasis. The results are shown in **Figure 3**. The AUC of bone metastasis was 0.724 (see **Figure 3A**), the AUC of brain metastasis was 0.717 (see **Figure 3B**), and the AUC of liver metastasis was 0.754 (see **Figure 3C**). These results suggested that the nomograms we constructed could accurately predict organ metastasis.

### 3.3 Nomogram predicting the survival probability of LC patients

**Figure 4:**
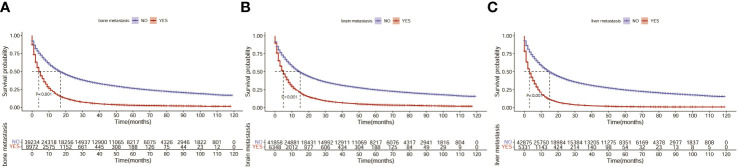
Survival of LC patients with distant metastasis. **(A)** Survival of LC patients with bone metastasis. **(B)** Survival of LC patients with brain metastasis. **(C)** Survival of LC patients with liver metastasis.

Next, we investigated the clinical factors affecting the prognosis of LC patients and attempted to construct a prognostic nomogram based on these clinical characteristics. KM analysis was used to show the survival of LC patients among the different subgroups (**Figure 4**). The median OS for all the patients in the whole cohort was 12 months. The median OS times of the patients with bone, liver and liver metastasis were 4, 5 and 3 months, respectively.

**Figure 5:**
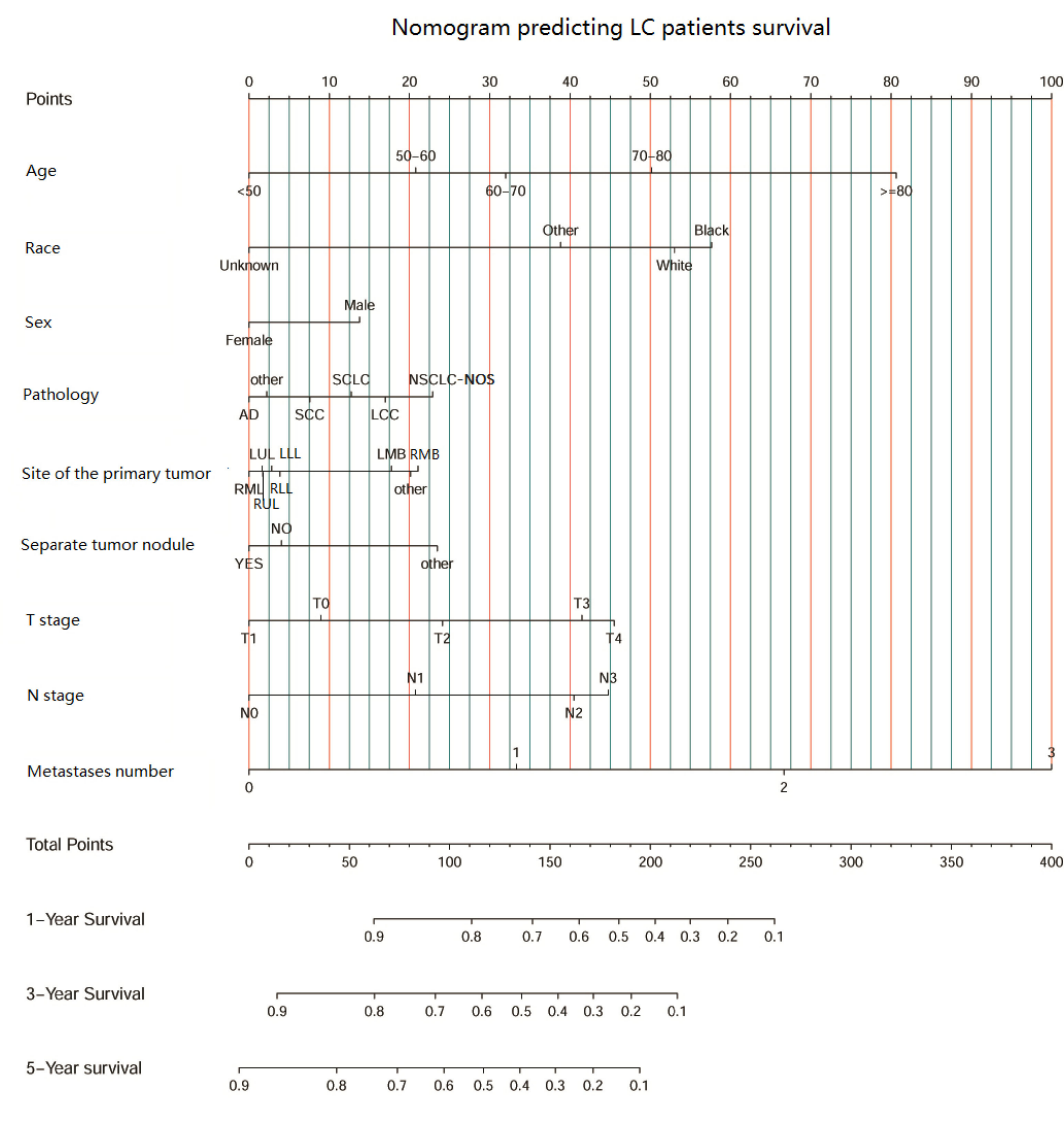
The nomogram predicting LC patient survival based on clinical factors.(SCC, squamous cell carcinoma; AD, adenocarcinoma; SCLC, small cell lung carcinoma; NSCLC-NOS, non-small cell lung carcinoma-not otherwise specified; LCC, large cell carcinoma; LLL, left lower lobe; LMB, left main bronchus; LUL, left upper lobe; RLL, right lower lobe; RMB, right main bronchus; RMB, right middle lobe; RUL, right upper lobe.).

**Figure 6:**
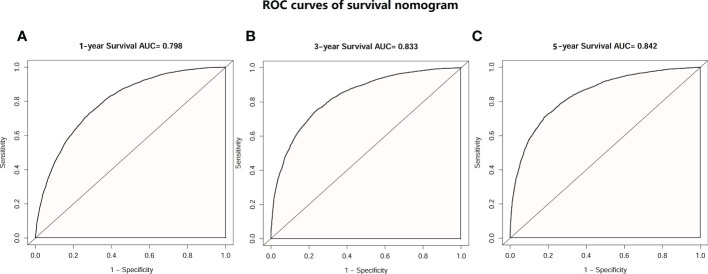
ROC curves of the survival nomogram. **(A)** ROC curve of 1-year survival prediction nomogram in patients with LC. **(B)** ROC curve of 3-year survival prediction nomogram in patients with LC. **(C)** ROC curve of 5-year survival prediction nomogram in patients with LC.

Then, univariate and multivariate Cox analyses were conducted to explore the potential prognostic factors. The results showed that age, sex, race, pathology, primary lesion site, separate tumor nodule, T stage, N stage, and number of organ metastases were all associated with the development of brain metastasis. The Cox analysis results are shown in **Table 4**. All prognostic factors were used to construct a nomogram predicting the survival of LC patients at 1, 3, and 5 years based on the training set (see **Figure 5**). The c-index of this nomogram was 0.719 (95% CI, 0.715-0.723) for the training set and 0.718 (95% CI, 0.714-0.722) for the test set. Then, we constructed ROC curves to evaluate the accuracy of the nomogram in predicting the 1-, 3- and 5-year survival probabilities in the test set (**Figure 6**). The AUC of 1-year survival was 0.798, 3-year survival was 0.833 and 5-year survival was 0.842. These results suggested that the nomogram had good predictive performance for LC patient survival.

**Table 4:** Univariate and multivariate cox analysis results of the prognostic factors LC patients.

	Univariate cox analysis		Multivariate cox analysis	
	HR (95%CI)	P value	HR (95%CI)	P value
Age				
<50	1 (reference)	1 (reference)	1 (reference)	1 (reference)
50-59	1.29(1.22-1.37)	<0.001	1.36(1.28-1.44)	<0.001
60-69	9(1.31-1.47)	<0.001	3(1.54-1.73)	<0.001
70-79	61(1.52-1.70)	<0.001	2.15(2.03-2.28)	<0.001
>=80	2.25(2.12-2.39)	<0.001	3.46(3.27-3.67)	<0.001
Race				
Black	1 (reference)	1 (reference)	1 (reference)	1 (reference)
White	0.98 (0.94-1.01)	0.1910	0.94(0.90-0.97)	<0.001
Other	0.91 (0.87-0.95)	<0.001	0.77(0.74-0.81)	<0.001
Unknown	0. 35(0.24-0.50)	<0.001	0.45(0.31-0.65)	<0.001
Sex				
Female	1 (reference)	1 (reference)	1 (reference)	1 (reference)
Male	1.3(1.27-1.33)	<0.001	1.23(1.21-1.26)	<0.001
Pathology				
AD	1 (reference)	1(reference)	1 (reference)	1 (reference)
SCC	(1.05-1.11)	<0.001	1.13(1.10-1.16)	<0.001
LCC	(1.11-1.31)	<0.001	1.29(1.19-1.41	<0.001
NSCLC-NOS	(1.48-1.60)	<0.001	38(1.33-1.44)	<0.001
SCLC	1.72(1.67-1.77)	<0.001	1.22(1.18-1.26)	<0.001
Other	0.75(0.73-0.77)	<0.001	1.02(0.99-1.06)	0.1268
Site of the primary tumor				
LLL	1 (reference)	1 (reference)	1 (reference)	1 (reference)
LMB	65 (1.53-1.78)	<0.001	1.18(1.10-1.28)	<0.001
LUL	1.04 (1.00-1.07)	0.0551	0.99(0.95-1.03)	0.5721
RLL	1.04(1.00-1.08)	0.0580	1.03(0.99-1.07)	0.1550
RMB	1.88(1.76-2.01)	<0.001	1.31(1.22-1.40)	<0.001
RML	0.90(0.86-0.96)	<0.001	0.94(0.89-0.99)	0.0268
RUL	1.03(0.99-1.07)	0.1060	0.99(0.96-1.03)	0.6923
Other	1.85(1.77-1.93)	<0.001	1.31(1.26-1.37)	<0.001
Separate tumor nodule				
NO	1 (reference)	1 (reference)	1 (reference)	1 (reference)
YES	1.74(1.70-1.78)	<0.001	0.94(0.92-0.97)	<0.001
other	97(1.88-2.07)	<0.001	1.33(1.26-1.39)	<0.001
T stage				
T1	1 (reference)	1 (reference)	(reference)	1 (reference)
T2	1.80(1.75-1.86)	<0.001	1.45(1.40-1.49)	<0.001
T3	2.56(2.49-2.65)	<0.001	1.86(1.79-1.93)	<0.001
T4	18(3.08-3.28)	<0.001	1.99(1.92-2.06)	<0.001
TO	53(2.24-2.86)	<0.001	1.14(1.01-1.29)	0.0406
N stage				
NO	1 (reference)	1 (reference)	1 (reference)	1 (reference)
N1	66(1.60-1.72)	<0.001	41(1.36-1.46)	<0.001
N2	57(2.51-2.64)	<0.001	1.88(1.83-1.93)	<0.001
N3	2.90(2.81-2.99)	<0.001	1.99(1.93-2.06)	<0.001
Number of metastases	1.87(1.85-1.89)	<0.001	1.67(1.64-1.69)	<0.001

### 3.4 Prognostic value of the nomogram score

**Figure 7:**
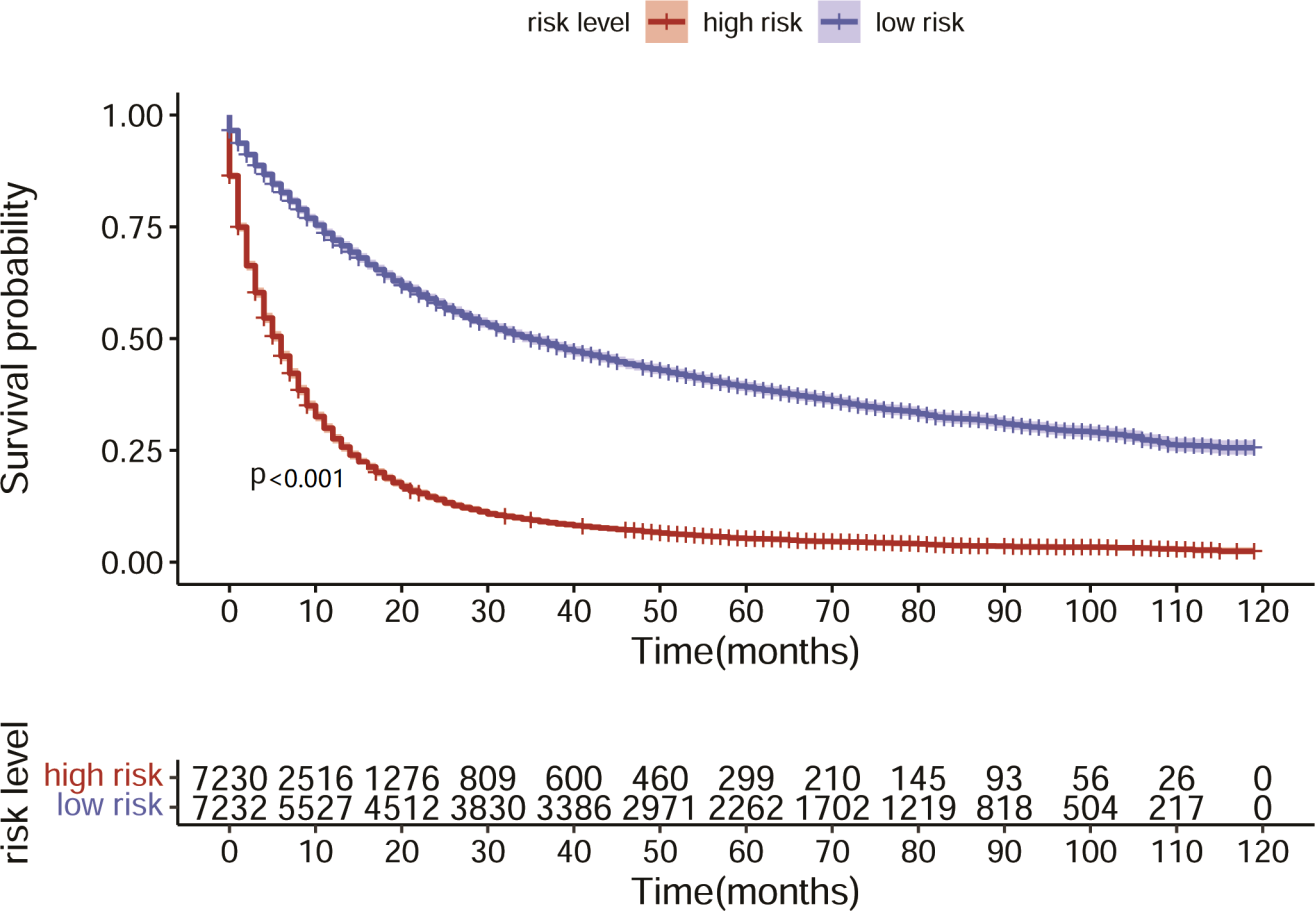
KM analysis showed survival outcome of high- and low- risk patients.

Based on the nomogram we constructed, we scored the patients in the test set and divided them into high- and low-risk groups according to the median nomogram score. The median survival time of the high-risk group was 6 months, while the median survival time of the low-risk group was 34 months. The KM analysis suggested that the survival difference between the high- and low-risk groups was significant (**Figure 7**). The patients in the high-risk group had shorter survival times. This result indicated that the predicted score of our model is closely associated with patient prognosis.

## 4 Discussion

As the second most common tumor, LC is a serious threat to human health. In recent years, with the development of comprehensive cancer treatments, including surgery, radiotherapy, traditional chemotherapy, targeted therapy and immunotherapy, the survival time of LC patients has been prolonged. However, distant metastasis of LC is still an obstacle to treatment and affects the survival of patients. Evaluating the possibility of developing distant metastasis based on clinical characteristics and examining patients at high risk to detect distant organ metastasis earlier could help physicians adjust treatment plans and improve the prognosis of patients.

Previous studies have suggested that SCLC usually metastasizes to the liver, bone, brain and other organs. Genetic changes may affect its metastatic site (17). In addition, the injection of SCLC cells into the middle vein of mice in one study specifically led to the occurrence of liver metastasis rather than lung metastasis (18). This suggests that small cell lung cancer cells may be more likely to metastasize to the liver through the blood, and the potential mechanism of its metastasis remains to be studied. For NSCLC, the AD and LCC pathological types are associated with a higher risk of brain metastasis than SCC (12, 19). Few studies have focused on the survival risk comparison among LC with different distant metastases and the prediction of distant metastases. In our study, our results showed that various pathologic types of LC show a strong correlation with site-specific metastasis patterns. Our results revealed that the most common metastatic organs are bone for SCC and AD, liver for SCLC and brain for LCC. SCLC is the most prone to distant organ metastasis, as well as multiple organ metastases, especially bone+liver metastasis, while SCC is the least prone. This is consistent with the results of a previous study (20). Regarding patient survival, a study focusing on NSCLC showed that adenocarcinoma is the most common variant for NSCLC, and the mortality risk is highest in multiple metastasis and liver metastasis groups (21). Similarly, in our study, the patients with triple metastases (brain-bone-liver) had the worst prognosis, and for nonsquamous carcinoma with single organ metastasis, liver metastases conferred the worst prognosis, which is consistent with the results of another study (22, 23). However, for SCC patients, there was no significant difference between the survival of SCC patients with single liver and single brain metastasis.

Approximately 30-40% of NSCLC patients will have bone metastasis (22, 24). Research has found that bone metastasis of LC is associated with the cytokines TGF-β and PTHrP, which can promote osteolysis (25–27). SCC can produce a large amount of MMP9 *via* stimulation by collagen I, thus establishing bone metastasis and releasing tumor cell chemokines during osteolysis (28). Our results suggest that SCC patients with bone metastasis alone have the worst prognosis compared with those with brain metastasis and liver metastasis alone. Two previous retrospective studies (29, 30) on esophageal and gallbladder cancer also reached similar conclusions; that is, among patients with single-organ metastasis, patients with bone metastasis had the worst prognosis (compared with patients with liver metastasis and lung metastasis). In addition, in a real-world study on patients with lung squamous cell carcinoma in 2022, the author found that only bone metastasis was found in distant organ metastasis, which was significantly related to the shorter PFS of these patients (31). This suggests that bone metastasis may be the special metastatic site of these tumors. Because few studies have compared the prognosis of different metastatic modes/sites of lung squamous cell carcinoma, the reason for this phenomenon is not clear. Its potential mechanism needs to be elaborated in future research.

We also investigated the risk factors for developing different organ metastases. LC patients of other races (American Indian/Alaska Native race) were more prone to brain and bone metastasis but not liver metastasis. A study exploring the risk factors for BM from esophageal cancer revealed that other races (American Indian/Alaska Native race) were positively associated with the occurrence of brain metastasis (32). All these results suggest that race is an important factor influencing tumor metastasis with unknown mechanisms. A previous study suggested that male sex is associated with a higher risk of brain metastasis in SCLC (33). According to our results, in addition to brain metastasis, male patients are also more likely to develop liver and bone metastasis. The logistic regression analysis showed that the characteristics of the primary tumor, including the location of the primary tumor, pathological type, T stage, N stage, and separate nodules, were closely related to the occurrence of distant metastasis. Compared with the left lower lobe, tumors with a primary focus in the center lung are less prone to organ metastasis, especially tumors with a primary focus in the upper and middle lobes of the center lung. Previous studies have reported that lung adenocarcinoma with a primary focus within the left side has a higher risk of skull metastasis than lung adenocarcinoma with a primary focus within the center side. Peripheral lung cancer was associated with brain metastasis (34), while central lung cancer was associated with bone metastasis (35). However, the research results of Mujoomdar et al. suggested that the risk of brain metastasis of NSCLC was not related to the location of the primary tumor (36). The relationship between the location of the primary lesion and distant metastasis has not been agreed upon. In addition, our results suggest that among various pathological types, patients with SCC are the least likely to have organ metastasis, patients with AD have a higher risk of bone metastasis and brain metastasis, and patients with SCLC have a higher risk of liver metastasis than patients with other pathological types. Patients with higher T stage, N stage and independent tumor nodules have a higher risk of bone, brain and liver metastasis, which is consistent with several previous studies (9, 13). Our nomograms based on these clinical factors showed good performance in predicting the occurrence of bone (AUC=0.724), brain (AUC=0.754) and liver (AUC=0.717) metastasis.

In this study, based on the multivariate Cox analysis, it was suggested that a primary lesion located in the main bronchus was a poor prognostic factor for LC patients, which caught our attention. Similar results were achieved in previous studies focusing on AD and LCC (37–39). The researchers found that the SUV value of tumors located in the center of PET was significantly higher, which may indicate that there was more active tumor metabolism (40). Main bronchial tumors are closely related to a higher risk of lymph node metastasis and organ metastasis (38, 41). In addition, patients with central tumors are more likely to develop obstructive pneumonia, which also leads to poor prognosis (42). These are all possible factors leading to poor prognosis of main bronchial tumors. In the future, more multicenter clinical studies are needed to reveal the potential mechanisms.

Based on the results of the Cox analysis, we established a survival nomogram to predict the survival of LC patients. The nomogram included age, race, sex, pathology type, primary site, whether there was a separate tumor nodule, T stage, N stage and the number of organ metastases. Although several prognostic models were established for patients with LC or NSCLC with brain metastasis in previous studies (13, 14, 43), separate tumor nodules, primary lesion sites and the number of organ metastases were included together as prognostic factors for the first time. Our model gives individual scores according to the clinical characteristics of each patient and predicts the 1-year, 3-year and 5-year survival rates. The model was independently verified in the test set, and the results showed a high concordance index of 0.718 (95% CI, 0.714-0.722) and an AUC score of 0.842. This suggests that our nomogram has a satisfactory predictive ability and shows better performance than the previous prediction models (37, 44, 45). The combination of the separate nodule status, the location of the primary lesion and the number of organ metastases makes this model more comprehensive and personalized to predict the prognosis and survival of different LC patients.

This study has some limitations. Not all the metastasis statuses of LC patients in the SEER database are clearly described. Therefore, the incidence of brain metastasis, liver metastasis and bone metastasis may be inaccurate. The SEER database does not provide detailed information about the follow-up treatment (including chemotherapy, immunotherapy, targeted treatment, etc.) that the patients received, which may contribute to potential bias and may influence the prognosis of patients, and follow up treatment was not included in this study. In addition, previous studies suggested that extrathoracic lymph node metastasis (46–48) was also a key factor affecting the prognosis of LC patients, but this variable is missing for the patients including in this cohort in the SEER database. This will be a key clinical factor for optimizing our predictive model in the following studies. In future research, more clinical data of stage IV LC patients with distant metastasis could be collected prospectively, as well as the therapy and survival time of these patients, so as to verify the results in this study in clinical data and test the accuracy and predictive performance of the model we constructed. What’s more, the molecular mechanisms underlying the preferential metastatic sites of different types of LC need to be elucidated in future studies.

## 5 Conclusion

In this study, we described a detailed landscape of distant organ metastasis statuses and their effects on overall survival in different pathological types of lung cancer. We found that the pathology of LC showed a strong correlation with site-specific metastasis patterns. Moreover, we investigated the risk factors for developing distant organ metastases in LC patients using data downloaded from the SEER database and constructed nomograms that predict distant metastasis and OS with good performance. These results are helpful for clinicians to conduct clinical evaluations and develop individualized therapeutic strategies.

## Data availability statement

The original contributions presented in the study are included in the article/**Supplementary Material**. Further inquiries can be directed to the corresponding author.

## Ethics statement

SEER database is publicly accessible worldwide. The authors signed the SEER database agreement and got the license to access SEER data.

## Author contributions

YH: research design, data collection, interpretation and analysis, manuscript drafting. GL: research design, critical manuscript revision. All authors contributed to the article and approved the submitted version.
